# A pilot trial investigating feasibility and preliminary efficacy of a task-specific step training regimen to improve balance recovery among community-dwelling older adults

**DOI:** 10.1371/journal.pone.0354677

**Published:** 2026-07-31

**Authors:** Youngjae Lee, Neil B. Alexander, Christopher T. Franck, Julia Castleberry, Michael L. Madigan

**Affiliations:** 1 Grado Department of Industrial and Systems Engineering, Virginia Tech, Blacksburg, Virginia, United States of America; 2 Department of Internal Medicine, Division of Geriatric and Palliative Medicine, University of Michigan, Ann Arbor, Michigan, United States of America; 3 Veterans Affairs Ann Arbor Health Care System Geriatric Research Education and Clinical Center, Ann Arbor, Michigan, United States of America; 4 Department of Statistics, Virginia Tech, Blacksburg, Virginia, United States of America; 5 School of Health Sciences, Emory and Henry University, Emory, Virginia, United States of America; Tokyo Metropolitan Institute of Geriatrics and Gerontology, JAPAN

## Abstract

While a growing number of studies have shown positive effects of perturbation-based balance training on balance recovery after tripping, this training has employed specialized equipment that may pose a barrier for wider adoption. To address this, the purpose of this pilot trial was to evaluate the feasibility and preliminary efficacy of a novel, low-resource (i.e., not requiring specialized equipment) version of perturbation-based balance training referred to here as task-specific step training. Thirty community-dwelling older adults (mean (SD) age: 71.8 (4.4) years) were recruited and allocated to either step training (*n* = 10), traditional treadmill perturbation-based balance training (*n* = 10), or a control group involving no training (*n* = 10). Participants were then exposed to two overground laboratory-induced trips while walking on a walkway. Results showed the step training group exhibited an initial recovery step that was 9.0% body height longer (*p* < 0.001) and 0.25 m/s faster (*p* = 0.011) than the control group.The step training group also exhibited a 4.8% body height longer recovery step, and a fall rate that was 39% lower (*p* = 0.037) when compared to the treadmill training group after lab-induced trips. While promising, these results should be interpreted with caution give the modest sample size and a potential bias between groups with respect to safety harness usage during laboratory-induced trips. A future trial with adequate statistical power to better evaluate efficacy and effectiveness of this step training on real-world trip and fall risk appears warranted. The study was registered on clinicaltrials.gov (NCT05734443).

## Introduction

Falls are the leading cause of injuries among adults aged 65 and over in the United States [1,2]. Many of these falls are thought to be due to an age-related decline in balance recovery ability [3–7]. Perturbation-based balance training (PBT) is a relatively new exercise-based fall prevention strategy that aims to train and improve this balance recovery ability. PBT exposes trainees to externally-applied postural perturbations in order to elicit balance recovery movements to avoid falling [8,9]. While the term PBT can be used regardless as to the nature of the external perturbations, task-specific PBT targets balance recovery after a specific type of perturbation such as tripping.

Tripping is the most common cause of falls among community-dwelling older adults [10–12]. Successful balance recovery after tripping has three requisites: 1) complete quick and sufficiently long recovery steps to extend the base of support beyond the body center of mass, 2) arrest trunk flexion, and 3) maintain adequate hip height for repeated stepping [4,13–16]. Several studies, including a recent review [17], have reported task-specific PBT to improve balance recovery and reduce the risk of falls after lab-induced trips or trip-like perturbations [13,18–25] or after real-world trips [26,27]. These studies utilized a specialized treadmill or other equipment unavailable the public to induce trip-like perturbations during PBT. An alternative PBT modality that still trains balance recovery after tripping, yet does not require specialized equipment, may help facilitate the wider adoption of PBT to reduce the risk of trip-induced falls [8,28].

A number of studies targeting fall prevention have investigated or proposed step training regimens that do not require a specialized treadmill or other equipment [29]. These regimens have included both volitional stepping movements often with targets on mats [30–32] and reactive stepping response to therapist-applied perturbations [33,34]. Evidence suggests such step training regimens can improve fall risk factors, clinical measures of mobility, and fall risk [29]. However, these step training regimens were not designed to train the specific balance recovery requisites after tripping, and may show greater benefits on trip-induced falls if designed to do so. An exception to this is where Ho et al. [35] reported on the clinical acceptability of a PBT program that uses both volitional stepping and reactive stepping to train balance recovery after trips and slips. The efficacy of this PBT program has yet to be reported.

The purpose of this pilot trial was to evaluate the feasibility and preliminary efficacy of a low-resource (i.e., not requiring specialized equipment) task-specific step training regimen that targets balance recovery after tripping. For comparison, separate groups of participants completed treadmill-based PBT (i.e., treadmill training group) or no training (i.e., control group). Feasibility was evaluated using recruitment rate and intervention adherence. Given the pilot nature of this trial, preliminary efficacy was evaluated using two hypotheses. First, we hypothesized that the step training group and treadmill training group would exhibit improved balance recovery and fall rates after laboratory-induced trips compared to the control group. Second, we hypothesized that the treadmill training group would exhibit more improved balance recovery and fall rate after laboratory-induced trips compared to the step training group. This second hypothesis was based on the expectation that the reactive stepping in response to sudden trip-like perturbations on a specialized treadmill would be more beneficial than the step training regimen under evaluation here that was based largely on volitional stepping [8,29]. Also given the pilot nature of this study, confidence intervals of group means and effect size indices were reported for the purpose of planning a larger study. If ultimately shown to be efficacious, this task-specific step training regimen could provide a more practical and accessible PBT method for targeting trip-induced falls and facilitate its wider application outside of the research environment.

## Materials and methods

A sample size of 30 (10 per group) was targeted and based upon resource availability. Participants were recruited from the university and local community via email listservs, flyers, word-of-mouth, and visits to local community organizations. Eligibility criteria required: community-dwelling individuals aged 65–80 years; body mass ≤ 114 kg due to safety harness weight limit; no lower limb amputation; no history of hip or vertebral fracture; no hospitalization within the last six months; no current back, leg, or foot pain that interfered with standing or walking; no dependency on an assistive device to walk; no regular participation in any kind of exercise to improve balance; score ≥ 19 on the Montreal Cognitive Assessment – Blind [36]; and bone mineral density *t*-score of the lumbar spine and proximal femur > −2.5 from Dual Energy X-ray Absorptiometry (Lunar iDXA, GE Healthcare, Chicago, IL). The study was approved by the Virginia Tech Institutional Review Board (#21–1072), and all participants provided written consent witnessed by a member of the research team (YL) prior to participation.

A three-group, parallel, posttest-only design was used. Participants were assigned to step training (*n* = 10), treadmill training (*n* = 10), or a control group (*n* = 10) using minimization [37,38] to balance groups with respect to age, sex, and unipedal stance time [39]. The same researcher (YL) conducted the minimization, assigned participants to groups, and assessed outcomes. Participants completed the Berg Balance Scale [40] at baseline to help characterize our sample. The step training group and treadmill training group completed two 45-minute training sessions per week for three weeks with each session involving 30 minutes of active training time. The control group completed no training. Three weeks after completing their assigned intervention, participants completed an outcome assessment session during which they were exposed to a laboratory-induced trip while walking overground, followed in later trials by a laboratory-induced slip and then a second trip, with multiple walking trials between each trip and slip. Participant recruitment took place from November 1, 2022 to June 26,2023, and all testing was completed by August 4, 2023. Due to an administrative oversight, the study was not registered on clinicaltrials.gov (NCT05734443) until February 2023. No changes to our methodology were implemented once participant recruitment started. Given that this study is a randomized pilot trial, the appropriate CONSORT reporting guidelines were used [41].

Details of the task-specific step training protocol have been reported elsewhere [42] (see Supplementary Material for a video that illustrates the protocol). It targets the requisites for balance recovery after tripping by emphasizing: 1) completing long, quick recovery steps to extend the base of support beyond the body center of mass, and 2) arresting trunk flexion. Each step training session began with a three-minute warm-up of walking on a treadmill and light stretching. It then proceeded through four phases of training involving repeated stepping trials at increasing levels of difficulty and with increasing similarity to actual trip recovery. *Phase 1 – Rapid Stepping* involved volitional stepping from bilateral standing where the participant self-initiated a forward fall by rotating forward about the ankles and then taking quick and long forward steps to recover balance. The participant was encouraged to fall as far forward as possible before starting to step, and to also take a long initial recovery step. This exercise was performed repeatedly first over a flat floor and then after placing a slender, lightweight foam obstacle with an 8.6 cm x 8.6 cm cross-section in front of their feet to promote stepping over an obstacle as is necessary during balance recovery after tripping. *Phase 2 – Trunk Control* involved similar stepping exercises as Phase 1 but with explicit instructions and emphasis on arresting forward trunk rotation by attempting to return the trunk to an upright posture at touchdown of the first recovery step. *Phase 3 – Lean Release* involved similar stepping exercises as Phase 2 but after an induced forward fall rather than a self-initiated forward fall. With the trainer standing in front of and facing the participant, the participant leaned forward by rotating about the ankle while being supported by the trainer with his/her hands on the participant’s shoulders. Without warning, the trainer released the participant and quickly stepped aside, while the participant took recovery steps to regain balance as in Phase 2. *Phase 4 – Simulated Trip* involved self-inducing a trip and then recovering using the elevating strategy [43]. The participant stood 15–30 cm away from an 8.6-cm-tall trip obstacle fixed to the floor, stepped toward the obstacle while purposefully impacting the front of the shoe on the obstacle to mimic a trip. The participant then allowed themselves to fall forward, then elevated the obstructed foot over the obstacle to complete their initial recovery step, and continued walking to establish a stable gait. As during Phases 1–3, Phase 4 was repeated while emphasizing taking a quick, long initial recovery step and controlling trunk posture to be vertical at touchdown of the first recovery step.

Step training was administered one-on-one and in person with the trainer (YL who was a graduate research assistant trained by MLM on the step training regimen) and with the difficulty of all phases individualized to each participant’s capability as perceived by the trainer and rate of improvement to continuously challenge the participant. To prevent a fall in the event of an unsuccessful balance recovery, the trainer and a spotter stood in front of and to one side of the participant, respectively. Additionally, during the first training session, the participant wore a safety harness suspended from an overhead track to prevent accidental falls to the floor while becoming familiar with the training protocol and gaining confidence in performing these stepping exercises. Additional details of the step training are included in the Supplementary Materials.

The treadmill training has been reported earlier [19,20,22] and involved repeatedly exposing participants to perturbations while standing on a treadmill (Freemotion 800, Freemotion Fitness, Logan, UT, USA) modified by the investigators to elicit sudden increases in belt speed. The participant first stood on the treadmill with the belt stationary. To elicit a trip-like forward loss of balance, the treadmill belt was accelerated posteriorly without warning to change the treadmill belt speed from 0 m/s to up to 1.07 m/s (i.e., 2.4 mph) in approximately 40 msec. The participant was instructed to react naturally and try to establish a stable gait after the change in belt speed. The belt speed was maintained until either the participant established a stable gait for several strides or the participant received substantial support from a safety harness or a spotter. Each treadmill training session involved up to 40 treadmill perturbations with a 5-minute break after 20 perturbations. Additional details of the treadmill training are available in the Supplementary Material.

The outcome assessment session was completed three weeks after the completion of step or treadmill training or, in the case of the control group, three weeks after the participant’s first visit to our laboratory. Two laboratory trips were induced while walking using methods described elsewhere [19,20,44]. Participants first completed a minimum of 10 walking trials on a 12-meter level walkway where a pneumatic trip obstacle was concealed and level with the walkway surface. Participants were asked to walk at a self-selected purposeful speed (i.e., as if they were going somewhere) while looking straight ahead. To interject some unexpectedness with respect to tripping, participants were informed that they may or may not be exposed to one or more unexpected trips or slips while walking, and if so, simply react naturally and continue walking. After completing a minimum of 10 walking trials, the trip obstacle was activated and quickly rose to a height of 8.6 cm without warning at the start of the stance phase of the non-dominant foot to attempt to trip the dominant foot during its ensuing swing phase. After the first trip, participants were exposed to a laboratory slip (the focus of a separate study reported elsewhere [45]) and then a second trip, each after a minimum of three walking trials from the prior perturbation. Participants wore standardized footwear (New Balance Athletics, Inc.) and safety harness to prevent an accidental fall to the floor in the event of an unsuccessful balance recovery. The harness was attached to an overhead track with the length of lanyard adjusted so that the participant’s knees, when asked to kneel while allowing the harness to fully support the body weight, were approximately 10 cm from the walkway surface.

Body position was sampled at 128 Hz during trip trials using a 13-camera motion capture system (Qualisys North America, Inc., Buffalo Grove, IL, USA) with 13 reflective markers placed at acromion processes, xiphoid process, spine at the same height as xiphoid process, sacrum, greater trochanters, lateral malleoli, and anterior/posterior tip of the shoes, and subsequently low-pass filtered at 10 Hz (fourth-order, zero-phase-lag, Butterworth filter). Force applied to the safety harness was sampled at 1280 Hz using a uniaxial load cell (Cooper Instruments, Warrenton, VA), and subsequently low-pass filtered at 40 Hz (fourth-order, zero-phase-lag, Butterworth filter). Data processing was then performed using custom code in MATLAB R2021a (The MathWorks Inc., Natick, MA, USA).

The balance recovery measures included: trunk angle at touchdown (of the first recovery step over the obstacle), recovery step length (of the first recovery step over the obstacle in anterior-posterior or AP direction), recovery step speed (mean AP speed of the first recovery step over the obstacle), distance from pelvis to stepping toe at touchdown (of the first recovery step over the obstacle in AP direction), and sacrum height at touchdown (of the first recovery step over the obstacle). In addition, gait speed, stepping strategy (elevating or lowering strategy), and trip outcome were determined. Trip outcome was classified by an investigator not blinded to group assignment as either a fall, recovery, harness-assist, or missed trip. A fall occurred if a participant was fully and continuously supported by the harness as observed from video. A recovery occurred if the integrated harness force from trip onset to one second after touchdown did not exceed 20% body weight * second. A harness-assist occurred if a trip was neither a fall nor recovery. A missed trip occurred if the leading edge of the swing foot did not contact the trip obstacle due to improper timing of triggering the trip obstacle. Additional details about the balance recovery measures are available in the Supplementary Material.

A mixed-model analysis of variance (ANOVA) with *a priori* pairwise contrasts was used to investigate differences in balance recovery measures from each trip between groups and included additional factors of subject identification number (as a random effect), trip number, stepping strategy, gait speed, and Berg Balance Scale score at baseline. Given the pilot nature of this study, the significance level of pairwise comparisons between the three groups was not adjusted for multiple comparisons. Body mass was explored as a covariate but ultimately not included due to lack of effects. Model output was inspected to visually confirm normally distributed residuals and homogeneity of variance across the groups. Power and log transformations of the balance recovery measures were used when needed for residuals of the ANOVA model to approximate a normal distribution with results back-transformed to the original scale for reporting. Outcome variables requiring transformations included: recovery step length, recovery step speed, distance from pelvis to stepping toe at touchdown, and sacrum height at touchdown. A Fisher’s exact test was used to investigate differences in trip outcome and stepping strategy between groups. Given the pilot nature of this study, group means and standard deviations, 95% confidence intervals, and 80% confidence intervals of each balance recovery measure were reported. Also reported were the 95% and 80% confidence intervals of Hedge’s *g* [46], which is a correction to Cohen’s *d* that improves accuracy when using small sample sizes (i.e., *n* < 20 per group). As noted earlier, feasibility was evaluated using recruitment rate and intervention adherence. JMP Pro 16 (SAS Institute, Inc., Cary, NC) was used for statistical analyses, effects were deemed statistically significant when *p* ≤ 0.05, and non-significant effects that approached significance *p* ≤ 0.10 were also noted.

## Results

**Fig 1:**
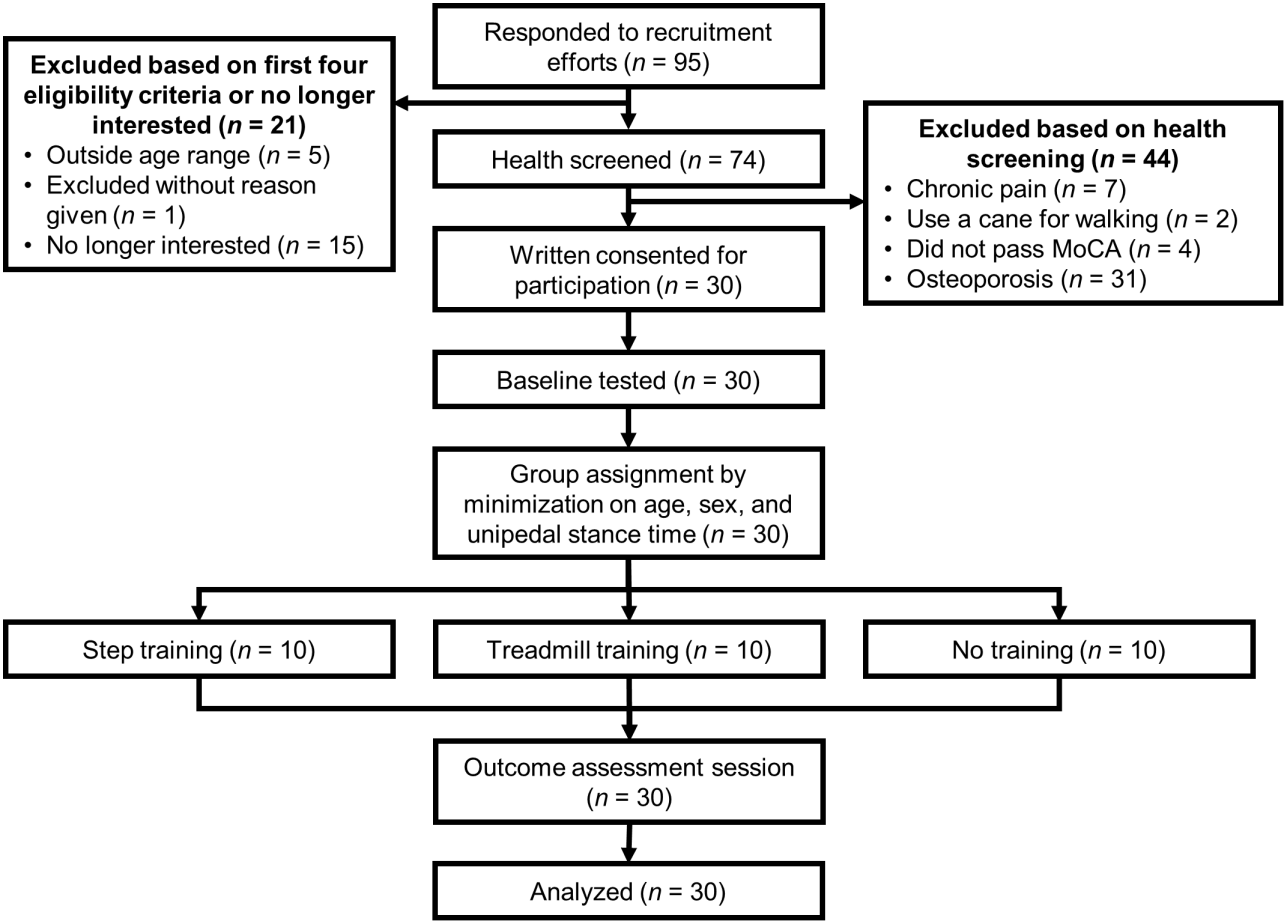
Protocol flow diagram. Number of participants involved throughout the study including recruitment, screening, group allocation, training, and outcome assessment. MoCA = Montreal Cognitive Assessment.

A total of 95 individuals responded to our recruitment efforts (Fig 1) with *n* = 21 being excluded during a telephone screening based upon inclusion criteria or loss of interest. The health screening excluded *n* = 44 participants, resulting in *n* = 30 participants, or 32% of the initial 95 individuals, being assigned to a treatment group (Table 1; 12 M and 18 F; mean (SD) age: 71.8 (4.4) years; height: 1.68 (0.10) m; mass: 77.1 (16.8) kg; and unipedal stance time:17.6 (12.3) s). Intervention adherence was 100% in that all 30 participants successfully completed their assigned six training sessions within three weeks. Three of the participants required moving one training session to a different week to accommodate scheduling constraints. The remaining 27 participants completed two training sessions per week for three weeks. All participants completed the outcome assessment session three weeks after completing training. The trial was ended based upon reaching our recruitment goal.

**Table 1:** Participant characteristics for each group.

	Step training (*n* = 10)	Treadmill training (*n* = 10)	Control (*n* = 10)	*p*-value^a^
**Sex (male/ female)**	4/ 6	4/ 6	4/ 6	–
**Age (years)**	71.9 (4.1)	71.8 (5.2)	71.8 (4.3)	0.998
**Unipedal stance time (s)**	18.9 (11.5)	16.5 (12.7)	17.3 (13.8)	0.917
**Height (m)**	1.63 (0.08)	1.69 (0.10)	1.70 (0.12)	0.277
**Mass (kg)**	69.9 (17.0)	79.6 (15.8)	81.9 (16.8)	0.244
**# of participants reporting one or more falls over prior six months**	1	3	1	–
**Berg Balance Scale**	55.1 (1.3)	54.4 (2.4)	54.1 (2.5)	0.441

^a^One-way analysis of variance with group as the independent variable.

**Fig 2:**
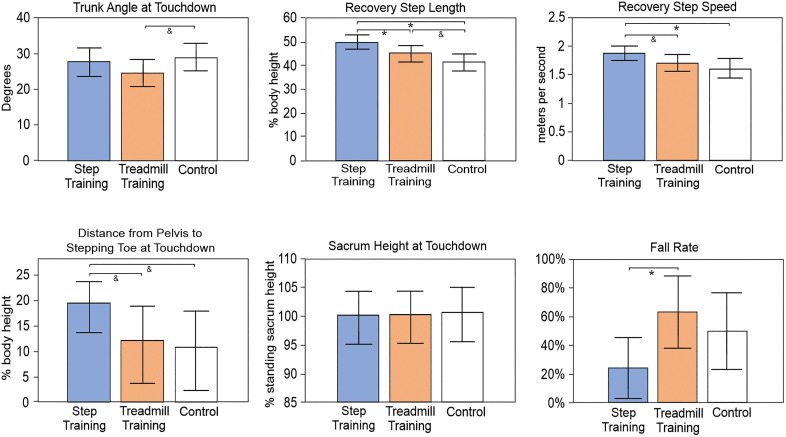
Model-based means and confidence intervals of balance recovery measures for each intervention group. Error bars represent 95% confidence intervals. * indicates *p* ≤ 0.05 and ^&^ indicates non-significant trends with *p* ≤ 0.10.

**Fig 3:**
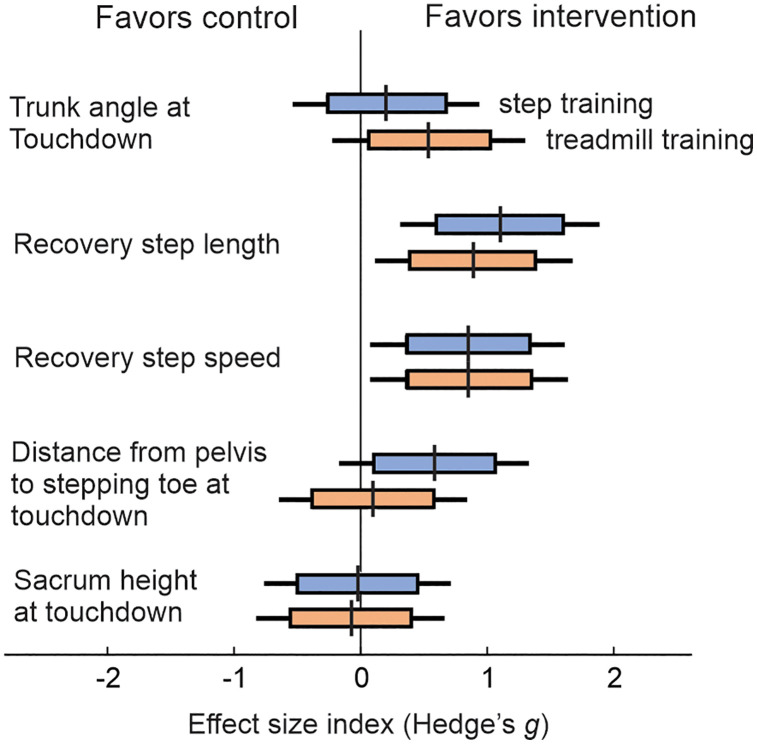
Confidence intervals of effect size index for balance recovery measures. Colored bars illustrate 80% confidence intervals of the effect size index (Hedge’s *g*) and the thin whiskers represent the 95% confidence intervals of the effect size index. Effect size indices were determined relative to the control group.

The outcome of the 60 trips (two from each of the 30 participants) included 22 falls, 27 recoveries, 7 harness-assists, and 4 missed trips (see Table A in S5 File for breakdown by group). Harness-assists and missed trips were excluded from further analysis. Regarding differences between Trip 1 and Trip 2, distance from pelvis to stepping toe at touchdown was 5.4% body height (BH) longer after Trip 2 than Trip 1 (*p* = 0.034) and sacrum height at touchdown was 4.1% sacrum height (SH) higher after Trip 2 than Trip 1 (*p* < 0.001). The remaining balance measures did not differ between Trip 1 and Trip 2 including trunk angle (*p* = 0.223), recovery step length (*p* = 0.443), and recovery step speed (*p* = 0.209; see Table B in S5 File for additional information). Several balance recovery measures differed between groups (Fig 2). When comparing the step training group to controls, the step training group exhibited an initial recovery step that was 9.0%BH longer (*p* < 0.001) and 0.25 m/s faster (*p* = 0.011), as well as an 8.4%BH longer distance from pelvis to stepping toe at touchdown than controls that approached significance (*p* = 0.055; Fig 2). When comparing the treadmill training group to controls, the treadmill training group exhibited a 4.7 deg smaller trunk angle at touchdown (*p* = 0.076) and 4.2%BH longer recovery step (*p* = 0.089) that both approached significance (Fig 2). When comparing the step training group to the treadmill training group, the step training group exhibited a 4.8%BH longer recovery step than the treadmill training group (*p* = 0.032), as well as a 0.15 m/s faster recovery step (*p* = 0.084) and 7.5%BH larger distance from pelvis to stepping toe (*p* = 0.073) that both approached significance (Fig 2). No other balance recovery measures differed between groups (*p* ≥ 0.247; Fig 2 and Table A in S4 File). 95% confidence intervals of Hedge’s *g* for recovery step length and recovery step speed favored both step training and treadmill training over controls (Fig 3). 80% confidence intervals of Hedge’s *g* for trunk angle at touchdown after treadmill training, and for distance from pelvis to stepping toe after step training, both also favored training over control (Fig 3).

The step training group exhibited a 39% lower fall rate (*p* = 0.037) than the treadmill training group, but neither of these groups exhibited a fall rate that differed from controls (*p* ≥ 0.157; Fig 2). The three groups exhibited no difference in stepping strategy used after laboratory-induced trips (*p* ≥ 0.688; Table S1). The mean (SD) gait speed across the included 49 falls or recoveries was 1.54 (0.21) m/s. No unintended harms to step training or treadmill training participants were identified. See Table C in S5 File for additional details on the ANOVA models.

## Discussion

The purpose of this pilot trial was to evaluate the feasibility and preliminary efficacy of a low-resource task-specific step training regimen. We employed a posttest-only experimental design and acknowledge its limitations including the inability to isolate training-induced effects from potential group differences that may have resulted despite our effort to balance groups using minimization. Acknowledging these limitations, we selected this design to mimic how step training would be implemented in practice (no pretest) and avoid the reduced external validity that can result from effects from a pretest. The limitations of our chosen study design, combined with the modest sample size of this pilot trial, may warrant a more cautious interpretation of between-group differences.

Regarding feasibility, 32% of 95 individuals who responded to our recruitment efforts were ultimately enrolled and exhibited 100% adherence for both step training and treadmill training. We used a safety harness during the first step training session to provide additional protection against falls beyond any support provided by the trainer and spotter. The cost and space requirements of this harness system could be a barrier to wider application of this step training. However, none of our participants fell into the harness at any point of the training. As such, we believe that future trials using similar populations could explore its application without the harness system while maintaining consistent vigilance and providing support, when needed, by the trainer and spotter.

Regarding preliminary efficacy, we first hypothesized that the step training group and treadmill training group would exhibit improved balance recovery and fall rates after laboratory-induced trips compared to controls. This hypothesis was partially supported as the step training group exhibited 9.0%BH longer and 0.25 m/s faster recovery step compared to controls. The treadmill training group exhibited 4.7 deg smaller trunk angle at touchdown and 4.2%BH longer recovery step compared to controls, but these only approached statistical significance. We also hypothesized that the treadmill training group would exhibit more improved balance recovery and fall rate after laboratory-induced trips compared to the step training group. This hypothesis was not supported because the step training group exhibited a 4.8%BH longer and 0.15 m/s faster recovery step, a 39% lower fall rate, and a larger distance from pelvis to stepping toe at touchdown compared to the treadmill training group 7.5%BH larger distance from pelvis to stepping toe that approached significance (Fig 2). Although preliminary, these trends suggest the low-resource task-specific step training improved balance recovery after laboratory-induced trips, and in some ways may provide greater beneficial effects than those elicited by treadmill-based PBT.. A larger follow-up study with a design and sample size informed by this pilot would have more statistical power to detect any such effects.

Although preliminary, trends within our data suggest step training may elicit greater improvements in stepping kinematics than treadmill training. The step training group exhibited a longer and faster initial recovery step compared to controls while the treadmill training group did not exhibit statistically significant difference from controls. Moreover, direct comparisons between step training and treadmill training showed the former to elicit longer and faster recovery steps and a larger distance from the pelvis to stepping toe. Among several studies that used a treadmill or overground obstacle for PBT targeting tripping and which compared stepping kinematics to a control group, none reported statistically significant improvements in step length [13,20,22,24] or step speed [22]. The step training investigated here involved a series of volitional and reactive stepping exercises during which participants were verbally instructed to emphasize a long and quick initial recovery step over a trip obstacle with steps up to approximately 1.1 m observed (motion capture was not collected during either training). The treadmill training group was not given the same instruction due there only being 0.6 m distance from the toes during the initial standing position on the treadmill to the front edge of the treadmill belt. While the sudden posterior movement of the treadmill belt increased this distance by the time the initial recovery step was completed, the length of the treadmill belt may have limited step length during training and potentially any training benefits on step length during treadmill training. It is also possible that the explicit verbal instruction and emphasis used here during step training to complete a long and quick initial recovery step may helped to improve stepping in response to the lab-induced trip. The longer and quicker initial recovery step after step training also generally aligns with prior studies that showed improved reaction and movement times on several clinical measures such as simple reaction time and choice stepping reaction time after 8–12 weeks of volitional step training [32,47].

Although also preliminary, trends within our data suggest treadmill training may elicit greater improvements in trunk control than step training. The 4.7 deg smaller trunk angle at touchdown among the treadmill training group compared to the control group approached significance (*p* = 0.076) whereas the difference between the step training group and control group did not. Furthermore, the 4.7 deg smaller trunk angle among the treadmill training group, compared to controls, was nearly within the 6–34 deg range of effect sizes for trunk angle after similar treadmill training of one day up to four weeks among older adults [19,22,48]. Prior PBT studies that used a treadmill or overground obstacle to train balance recovery after tripping, and included a control group, reported better trunk control after PBT [13,19,22,24]. Perturbations during treadmill training differed from those during most of step training in that they were more abrupt, included higher speeds, and varied unexpectedly in speed and direction than step training. These differences may more strongly promote improvement in trunk control after treadmill training than step training that involved less abrupt and self-imposed perturbations.

Although it was not statistically significant (*p* = 0.157; Fig 2), the fall rate among the step training group (24%) was approximately half of the fall rate among the control group (50%). We were unable to find any studies that evaluated step training using lab-induced trips so a direct comparison with the literature was not possible. A systematic review and meta-analysis by Okubo, Schoene [29] reported a similar 43% reduction in real-world falls among older adults across three studies [30,32,49] that investigated 12–24 weeks of volitional step training with stepping targets (e.g., mats partitioned into squares, colored stepping squares) when compared to active controls (e.g., walking, strength and balance exercise). Our smaller sample size than the meta-analysis reported by Okubo, Schoene [29] may have been insufficient to achieve statistical significance. The six sessions of step training over three weeks used here was a smaller training dosage than these three studies. It may also be noteworthy that our outcome assessment session during which participants were exposed to a laboratory-induced trip was three weeks after training and thus involved retention in addition to the initial training-based improvement.

Unexpectedly, although not statistically significant, the 63% fall rate among the treadmill training group was higher than the 50% in the control group (*p* = 0.722; Fig 2). Potentially related to this, the 24% fall rate among the step training group appears superior to the 63% fall rate among the treadmill training group (*p* = 0.037). At least two potential explanations exist for the elevated fall rate among the treadmill training group. First, despite our efforts to balance individual characteristics across the three groups using minimization, it is possible that the groups differed in important intrinsic fall risk factors that were not measured. Second, some treadmill training participants may not have used their best effort to recover their balance after laboratory-induced trips. A safety harness was used during all six sessions of treadmill training but only during the first session of step training and during no sessions of control training (since there was no control training). Moreover, some treadmill training participants experienced falling into the harness during their training while none of the step training or control training participants did. This may have resulted in a bias between groups in the perceived safety or risk with some treadmill training participants being less anxious about falling into the harness and, inadvertently, reducing their motivation to prevent it from happening. We also note, retrospectively, that their instructions were not to do their best to prevent a fall, but simply react naturally and continue walking if exposed to a trip. This may not have explicitly motivated participants to use their best effort to avoid falling into the safety harness. As such, the trends in the data that suggest the superiority of step training over treadmill training in terms of fall rate should be interpreted with caution and further investigation is warranted.

This study had several limitations. First, the investigators who completed the data analyses were not blinded to the group allocation. Second, the sample size was based upon resources available rather than a formal sample size analysis. However, the data presented here can be used to more adequately plan and power future studies of the task-specific step training. Third, participants were healthy, community-dwelling, and relatively high-functioning (mean Berg Balance Scale of 54.6), and the results may differ among other populations. Fourth, the informed consent process as well as instructions provided to participants at the start of the outcome assessment session included a warning of potential trips or slips while walking. While this is not a threat to internal validity since all participants were provided with the same instructions, it may be a threat to external validity because such warning is not likely to be provided in the real world. Therefore, it is unclear how well responses to laboratory-induced trips would generalize to the responses to real-world trips.

## Conclusions

Although preliminary, the results of this pilot trial suggested a novel step training regimen improved stepping kinematics after a laboratory-induced trip. This regimen has the benefit of not requiring specialized equipment and as such may be more scalable to non-research settings such as physical therapy clinics, community centers, and health clubs. The feasibility and potential benefits of this step training on balance recovery warrant future study and could contribute to strategies aimed at reducing real-world trip and fall risk.

## Supporting information

S1 File DOI of a video of task-specific step training. (TXT)

S2 File Additional details of task-specific step training protocol. (PDF)

S3 File Additional details of Methods. (DOCX)

S4 File Supplementary Results. (XLSX)

S5 File Data for sharing. (XLSX)
