# Supplementary material for: A pilot trial investigating feasibility and preliminary efficacy of a task-specific step training regimen to improve balance recovery among community-dwelling older adults
Source: PLoS One. 2026 Jul 31;21(7):e0354677. doi: 10.1371/journal.pone.0354677 (PMC13426968; doi:10.1371/journal.pone.0354677)
Supplement: S2 File — (PDF) [file pone.0354677.s002.pdf]

Task-Specific Step Training Protocol

Warm-up

- ☐ Ask if the subject how he/she is feeling and if he/she has any abnormal pain in legs or anything
- ☐ Check whether the subject is wearing appropriate shoes and clothes
- ☐ Ask to empty pockets and remove jewelry (place them nearby where visible)
- ☐ 3 minutes of treadmill walking in a comfortable speed
- ☐ Stretching – quadriceps, hamstrings, calves, lunges

Training Preparation

- ☐ *First session only*: Demonstrate what a trip is. Tell the subject that "your movements in the short period of time immediately after the trip are critical to preventing a fall. The goal of this training is to improve these movements. Some of which might sound strange, but there is a method to our madness."
- ☐ Set the 30-min timer and reference it when needed so that the active training time is 30 minutes.

Phase 1A – Rapid Stepping with no obstacle

- ☐ Demonstrate, with no obstacle
  - Subject stands on the wooden platform, starts to fall forward by dorsiflexing at the ankles and keeping the rest of the body straight, and then takes quick steps to recover balance
    - o Instruct to step first with their dominant foot (chosen foot to kick a ball as far as possible).
    - o Make sure to practice with both feet (i.e., not necessarily to go LRLR, but aim for a similar # of L or R) for an initial step. Complete at least five trials with each foot stepping initially.
  - Stand in front of the subject to provide support, if necessary
- ☐ Repeat and encourage subject to (1) wait as long as possible after starting to fall and before taking an initial recovery step and (2) take a longer initial recovery step. You may need to encourage each of these separately and/or gradually as their skill improves over the training sessions.

Phase 1B – Rapid Stepping with obstacle

- ☐ Repeat Phase 1A, but using the obstacle (Demonstrate first)

Phase 2A – Trunk Control with no obstacle

- ☐ Repeat Phase 1A, but with an emphasis on extending the back during stepping so that the trunk is upright at touchdown of the first recovery step (Demonstrate first)

Phase 2B – Trunk Control with obstacle

- ☐ Repeat Phase 2A, but using the obstacle (Demonstrate first)

Phase 3A – Lean Release with no obstacle

- ☐ Demonstrate, with no obstacle
  - Subject stands on the platform while trainer stands facing the subject and places hands on shoulders of the subject.
  - Subject leans forward by dorsiflexing at the ankles and while being supported by trainer (adjust the lean angle based on subject's performance)
  - When the trainer releases the subject without warning and steps away, the subject takes quick steps to recover balance.
    - o Instruct to step first with the dominant foot.
    - o Make sure to practice with both feet (i.e., not necessarily to go LRLR, but aim for a similar # of L or R) for an initial step. Complete at least five trials with each foot stepping initially.

- Be ready to provide support, if needed by the subject

☐ Make sure to release the subject from the lean without any cues so the perturbation timing cannot be anticipated
  - At some point, maybe even give anticipatory actions on purpose, but don't release
  - When the subject uses RIGHT (or LEFT) foot to recover, trainer steps to the RIGHT (or LEFT) upon the release

☐ Repeat the exercise and encourage to (1) take a longer initial recovery step and (2) have the trunk upright at touchdown of the first recovery step. You may need to encourage each of these separately and/or gradually as their skill improves over the training sessions.

Phase 3B – Lean Release with obstacle

☐ Repeat Phase 3A, but using the obstacle (Demonstrate first)

Phase 4 – Simulated Trip (combine all the stepping skills trained above during a simulated trip)

☐ Use the obstacle to demonstrate the elevating strategy for balance recovery after tripping
  - Subject stands on the wooden platform, roughly one step away from the obstacle.
  - Subject steps and intentionally induces a trip (i.e., start to fall forward and wait as long as possible for "sensation of trip"). They then perform an elevating strategy to recover balance and continue walking.
  - Instruct to trip and step first with the dominant foot. Then, alternate the initial tripping/stepping foot.
  - Make sure to practice with both feet (i.e., not necessarily to go LRLR, but aim for a similar # of L or R) for an initial step. Complete at least five trials with each foot stepping initially.
  - Be ready to provide support, if needed by the subject

☐ Repeat the exercise and encourage to (1) wait as long as possible before taking an initial recovery step over an obstacle, (2) take a longer initial recovery step, (3) have the trunk upright at touchdown of the first recovery step, and (4) look forward and not at the obstacle (and stare at the wall, if necessary). You may need to encourage each of these separately and/or gradually as their skill improves over the training sessions.

Note: Phases 1B, 2B, and 3B (the phases involving the tripping obstacle) were skipped during the first training session. This was because we wanted to make sure the subject understood the basic movement requirements under less difficult conditions (without obstacle) before adding the obstacle and increasing the difficulty of the tasks.
