# Supplementary material for: A pilot trial investigating feasibility and preliminary efficacy of a task-specific step training regimen to improve balance recovery among community-dwelling older adults
Source: PLoS One. 2026 Jul 31;21(7):e0354677. doi: 10.1371/journal.pone.0354677 (PMC13426968; doi:10.1371/journal.pone.0354677)
Supplement: S3 File — (DOCX) [file pone.0354677.s003.docx]

Additional details about the treadmill training

The treadmill belt speed was both progressively increased and varied pseudo-randomly across perturbations to continue to challenge the participant as their performance improved while also maintaining unexpectedness in perturbation speed. In addition, sporadic trials with the belt accelerating forward to elicit a backward loss of balance from the standing position were included but excluded in the analysis. Starting in the second training session, a slender, lightweight foam obstacle with 8.6 x 8.6 cm cross-section was placed within 3-7 cm in front of the participant’s toes before each perturbation to elicit a step over an obstacle similar to that needed during an actual trip. Treadmill training was administered for one participant at a time, and the participant wore a full body safety harness to protect knees or hands from contact with the treadmill in the event of an unsuccessful balance recovery.

Additional detail about the task-specific step training

Some practical aspects of the step training are worth noting. First, we administered step training within a large laboratory, but anticipate the minimum necessary training area to be approximately 2 m wide by 4 m long. Second, we used a safety harness during the initial step training session and this could be viewed as a barrier for wider application because it would require either anchoring the harness to building infrastructure or use of a portable gantry. We note that no participants fell into the harness during step training, and as such we feel it is possible that future applications of this step training regimen may be able to forgo the harness and instead use a spotter and potentially more discretion when increasing perturbation severity. Third, while we administered step training one participant at a time, it could easily be administered in groups of two or three trainees. In fact, observing another individual complete the training, while also completing the training, may offer some training benefits [Hagedoorn et al, 2025]. Fourth, it will be important to investigate the clinical acceptability of this step training as done recently for a similar step training regimen also not requiring a specialized treadmill or equipment [Ho et al., 2024].

Hagedoorn, L., et al., Action observation with motor simulation of reactive stepping: A randomized study in older adults with a history of falls. Exp Gerontol, 2025. 199: p. 112645.

Ho, C., et al., Clinician acceptability of the ReacStep reactive balance training program for fall prevention. Physiother Res Int, 2024. 29(4): p. e2133.

Additional details about the balance recovery measures

The balance recovery measures included: *trunk angle at touchdown* of the first recovery step over the obstacle (sagittal plane angle determined by a line connecting midpoint of the greater trochanter markers to the midpoint of the xiphoid process and spine markers, relative to quiet standing); *recovery step length* (anterior-posterior distance between the ankle marker of the non-stepping foot at trip onset and the ankle marker of the stepping foot at touchdown of the first recovery step over the obstacle); *recovery step speed* (anterior-posterior speed determined by dividing the recovery step length by recovery step completion time for the first recovery step over the obstacle); *distance from pelvis to stepping toe at touchdown* (anterior-posterior distance between the midpoint of the greater trochanter markers, as a proxy measure of center-of-mass, and toe marker at touchdown of the first recovery stop over the obstacle); and *sacrum height at touchdown* (vertical position of the sacrum marker at touchdown of the first recovery step over the obstacle). In addition, *gait speed* (anterior-posterior speed of the midpoint of the shoulder markers at trip onset), *stepping strategy* (elevating or lowering), and *trip outcome* were recorded. The trip outcome was classified as either a fall, recovery, harness-assist, or missed trip. A fall occurred if a participant was fully and continuously supported by the harness as observed from video. A recovery occurred if the integrated harness force from trip onset to one second after touchdown did not exceed 20% body weight * second. A harness-assist occurred if a trip was neither a fall nor recovery. A missed trip occurred if the leading edge of the swing foot did not contact the trip obstacle due to improper timing of triggering the trip obstacle.
